# A case of an intramesenteric GIST accurately diagnosed and completely resected by PET-CT and laparoscopy after gastrectomy for gastric cancer

**DOI:** 10.1093/jscr/rjac246

**Published:** 2022-06-16

**Authors:** Manabu Sato, Masayuki Sato, Tadaaki Yokoyama, Akiko Kusaka, Yukie Suzuki, Kenji Fukuhara

**Affiliations:** Department of Surgery, Shiogama City Hospital, Shiogama, Miyagi, Japan; Department of Surgery, Shiogama City Hospital, Shiogama, Miyagi, Japan; Department of Surgery, Shiogama City Hospital, Shiogama, Miyagi, Japan; Department of Surgery, Shiogama City Hospital, Shiogama, Miyagi, Japan; Department of Surgery, Shiogama City Hospital, Shiogama, Miyagi, Japan; Department of Surgery, Shiogama City Hospital, Shiogama, Miyagi, Japan

## Abstract

We performed the accurate diagnosis and complete surgical resection of a gastrointestinal stromal tumor at the mesentery of the small bowel. Computed tomography (CT) in a 62-year-old man at 2 years after gastrectomy for gastric cancer showed a mesenteric tumor, with no other tumors noted. Positron emission tomography-computed tomography (PET-CT) showed a maximum standardized uptake value (SUV max) of 2.9 at the tumor. The presence of a single and low SUV max tumor allowed us to perform laparoscopic surgery. Partial resection of the tumor with an adequate margin was performed. The pathological findings showed c-kit positivity and a low Ki-67 proliferation index (<5%). In the present case, PET-CT and laparoscopic assessments were useful for accurately evaluating the surgical resectability of the mesenteric tumor after distal gastrectomy for gastric cancer. The low SUV max and laparoscopic findings led to complete surgical resection of a mesenteric tumor.

## INTRODUCTION

Gastric cancer has a poor prognosis globally, and 40–60% of patients will suffer peritoneal relapse as the only site of recurrence after curative gastrectomy [1]. Therefore, intraperitoneal tumors detected by computed tomography (CT) and other modalities after a curative gastrectomy for gastric cancer are often diagnosed as metastatic peritoneal tumors. The prognosis of a metastatic peritoneal tumor is very poor, and there is no standard treatment [1, 2].

Gastrointestinal stromal tumor (GIST) is rare, with an incidence of 1–2% among gastrointestinal neoplasms [3]. The standard of treatment for GIST is complete surgical resection, and the prognosis after the complete resection is good, with an ≥80% relapse-free survival at 5 years in the low- and intermediate-risk groups [4, 5].

When an intraperitoneal tumor is detected by CT or other modalities after a curative gastrectomy for gastric cancer, the differential diagnosis is very important.

In the present case, we successfully performed laparoscopic resection of an intramesenteric tumor of the small bowel accurately diagnosed as a resectable tumor, not a metastatic tumor after the gastrectomy for gastric cancer.

## CASE REPORT

**Figure 1:**
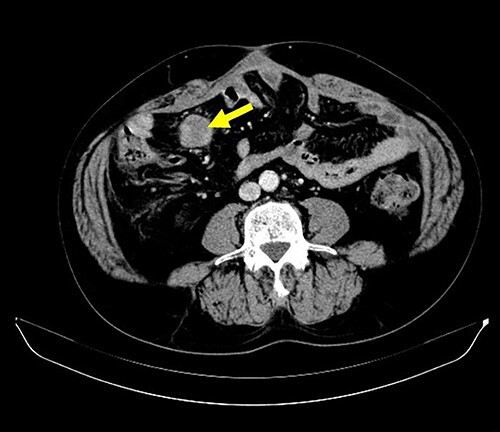
CT finding. Follow-up CT after gastrectomy for gastric cancer revealed a mesenteric tumor. There were no other malignant findings.

**Figure 2:**
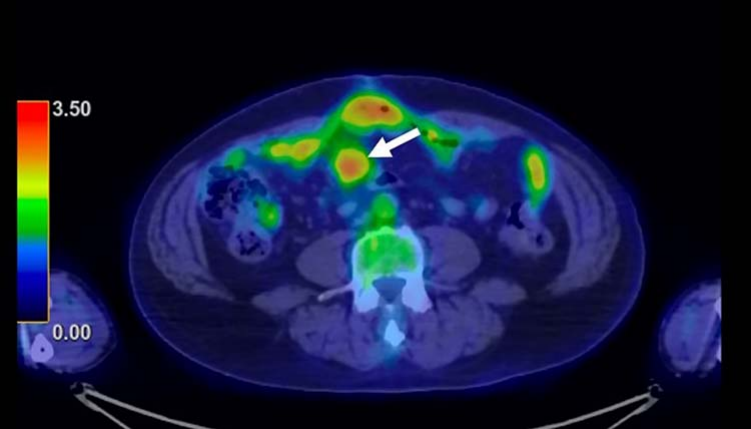
PET-CT finding. PET-CT showed that the maximum standardized uptake value (SUV max) of the mesenteric tumor was 2.9, with no other tumors present in the whole body.

A 62-year-old man underwent distal gastrectomy for gastric cancer. The pathological diagnosis was pT3N0M0 Stage IIA according to the TNM Classification of Malignant Tumors, 8th Edition. In our hospital, follow-up chest and abdominal CT are performed every 6 months for 5 years after gastrectomy. About 2 years later, follow-up CT scan revealed a mesenteric tumor. CT also showed the absence of the other evidence of recurrence of the gastric cancer, such as ascites, distant metastatic tumor and remnant gastric tumor (Fig. 1). The mesenteric tumor had not been identifiable on previous images after gastrectomy; thus, this image was the first image on which GIST was identified. Positron emission tomography (PET)-CT was performed to evaluate the mesenteric tumor and identify other tumors, showing that the maximum standardized uptake value (SUV max) of the mesenteric tumor was 2.9, with no other tumors present in the entire body (Fig. 2). The mesenteric tumor was deemed to be a resectable tumor such as a GIST, desmoid tumor or malignant lymphoma, and not peritoneal dissemination because CT and PET-CT revealed no other tumors.

**Figure 3:**
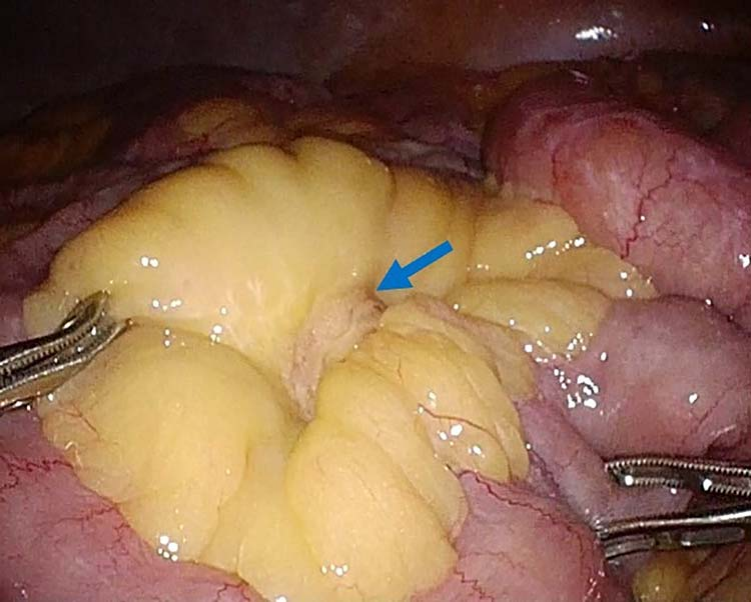
Laparoscopy finding. Laparoscopy showed a tumor in the mesentery of the small bowel, not arising from the bowel wall, with no obvious metastatic tumors of the liver or peritoneum.

Laparoscopic surgery was performed to diagnose and then resect the mesenteric tumor. The laparoscopic findings revealed that the tumor existed in the mesentery of the small bowel, and not arisen from the bowel wall, with no obvious metastatic tumors of the liver or peritoneum (Fig. 3). Subsequently, partial resection of the small bowel and mesentery with an adequate margin was performed.

**Figure 4:**
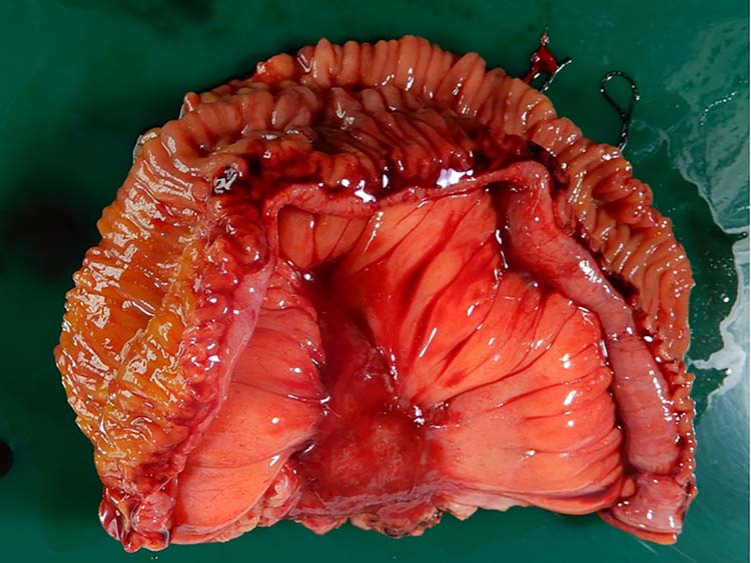
Macroscopic findings of the surgical specimen. Macroscopic findings revealed an intramesenteric tumor of the small bowel, not exposed to the abdominal cavity or the bowel lumen.

**Figure 5:**
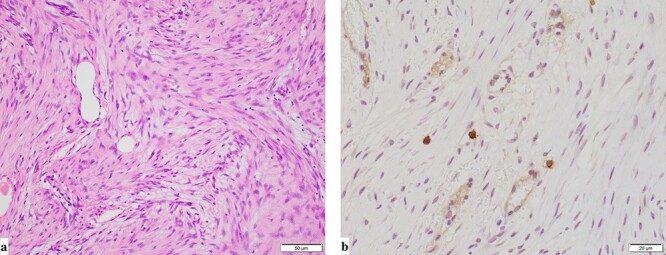
Pathological findings (**a**: hematoxylin and eosin staining, **b**: immunohistochemical staining for c-kit). Pathological findings showed that the mesenteric tumor was a GIST and c-kit-positive and CD34-, SMA- and S100-negative, with a low Ki-67 proliferation index (<5%).

The macroscopic evaluation of the surgical specimen revealed an intramesenteric tumor (45 × 35 × 20 mm in size) of the small bowel, without exposure to the abdominal cavity or the bowel lumen (Fig. 4). The postoperative course was uneventful. The pathological findings showed that the mesenteric tumor was a GIST that was c-kit-positive and CD34-, SMA- and S100-negative, with a low Ki-67 proliferation index (<5%) (Fig. 5). Because the tumor was considered to have a low risk of recurrence (<5 cm, low Ki-67 index, and not ruptured), adjuvant therapy was not performed. At 10 months after surgery, he remains free from recurrence of the GIST or gastric cancer.

## DISCUSSION

Complete surgical resection is the standard treatment for a localized GIST, and the relapse-free survival and overall survival rates are good after the complete resection, especially in low- or intermediate-risk group [3, 4, 6]. It is important to make an accurate differential diagnosis of GIST for the complete resection. However, GISTs are sometimes asymptomatic until the advanced stages because of their submucosal localization and noninvasive behavior, so an accurate diagnosis is often difficult [7]. In the present case, the intramesenteric GIST was asymptomatic and diagnosed by the follow-up CT after the gastrectomy for gastric cancer.

While the mesenteric tumor was detected by the follow-up CT, the differential diagnosis between GIST and a metastatic tumor of gastric cancer was very important, as the treatment strategies for these tumors are very different. The prognosis is very poor for metastatic tumors of gastric cancer, and treatments for recurrent gastric cancer include systemic chemotherapy, cytoreductive surgery with hyperthermic intraperitoneal chemotherapy and palliative treatment [1, 2, 8, 9]. In contrast, the prognosis is good after the complete resection for GISTs [3–6]. The relapse-free survival is good after the complete resection of GISTs with a low or intermediate risk of recurrence [4, 10].

In the present case, PET-CT was particularly helpful for distinguishing GIST from recurrence after gastrectomy for gastric cancer. Discussions about the differences in the SUV max on PET-CT between GISTs and metastatic tumors of gastric cancer are rare. Fluorodeoxyglucose-PET is reportedly useful for diagnosing recurrent gastric cancer, and the median SUV max of recurrent disease was ≥4.0 [11, 12]. However, Iwamura *et al*. reported that the SUV max of GISTs with a low mitotic index was significantly lower (2.4 ± 4.2) than that of GISTs with a high mitotic index (9.6 ± 7.6), indicating that the SUV max reflects the cell proliferation of GISTs [13]. These studies suggest that the SUV max of GIST with a low mitotic index might be lower than that of metastatic tumors of gastric cancer.

In the present case, the SUV max of the mesenteric tumor was 2.9, and there was no other metastatic tumor in the whole body. The low SUV max and presence of a single tumor indicated that tumor was a resectable tumor. After the diagnosis, we performed laparoscopic surgery. Observation with a laparoscope showed a mesenteric tumor at the small bowel, with no metastatic tumor. In advanced gastric cancer, staging laparoscopy is frequently performed to assess the gastric tumor and metastatic lesions, particularly in cases with peritoneal deposits [14]. Staging laparoscopy avoids unnecessary laparotomy and the morbidity of the laparotomy is minimal. In the present case, laparoscopic surgery was useful for estimating the resectability of the tumor and checking for peritoneal dissemination and other metastatic tumors.

## CONFLICT OF INTEREST STATEMENT

The authors declare that they have no interest.

## FUNDING

The authors received no funding for this study.

## AUTHORS’ CONTRIBUTIONS

Manabu Sato drafted the manuscript. K.F. and Masayuki Sato supervised the writing of the manuscript. Manabu Sato, Masayuki Sato, T.Y., A.K. and Y.S. provided managements of the patient. All authors read and approved the final manuscript.

## CONSENT FOR PUBLICATION

Written informed consent was obtained from the patient for the publication of the case and all accompanying images.
